# Small Bowel Stricture in a Crohn's Patient: An Unrelated Etiology

**DOI:** 10.1155/crgm/6697889

**Published:** 2025-06-24

**Authors:** Dhiraj K. Peddu, Matthew Kubina, Ankit Mishra, Molly Stone, Winnie Zou, Jiaqi Shi, David C. Kestenbaum, Scott E. Regenbogen, Jeffrey A. Berinstein

**Affiliations:** ^1^Department of Gastroenterology and Hepatology, Michigan Medicine, Ann Arbor, Michigan, USA; ^2^Department of Pathology and Clinical Labs, University of Michigan, Ann Arbor, Michigan, USA; ^3^Department of Interventional Radiology, Montefiore New Rochelle Hospital, New Rochelle, New York, USA; ^4^Department of Colorectal Surgery, Michigan Medicine, Ann Arbor, Michigan, USA

## Abstract

Small bowel strictures are a common complication of Crohn's disease (CD), which can lead to obstruction, perforation, and fistula formation. However, strictures can stem from other etiologies in CD patients, including malignancy, prior surgery, radiation, and ischemia. We present a patient who developed a new long-segment jejunal and ileal stricture within 2 months after ileocolic resection. What was initially treated as worsening CD was ultimately an unrelated ischemic stricture due to suspected superior mesenteric artery thrombosis following ileocolic resection. The contrasting location of the stricture compared to her previous disease, timing of progression, and lack of response to anti-inflammatory treatment prompted a reassessment of the underlying disease process.

## 1. Introduction

Crohn's disease (CD) is an autoimmune condition that can cause inflammation throughout the gastrointestinal tract resulting in abdominal pain, and frequent bowel movements, and can lead to stricture, fistula, and abscess formation [1]. While patients can have progression in CD phenotype and severity, the location of the disease often remains stable [2, 3]. While new areas of the disease can develop outside of a patient's typical distribution, it is imperative to consider alternative explanations rather than automatically attributing them to a patient's existing CD diagnosis. This is especially true when minimal response to anti-inflammatory agents is observed [2]. We present a patient with long-standing stricturing short-segment ileocolonic CD treated with ileocolic resection, who subsequently developed new long-segment jejunal and ileal inflammation and stenosis unrelated to her CD.

## 2. Case Report

**Figure 1:**
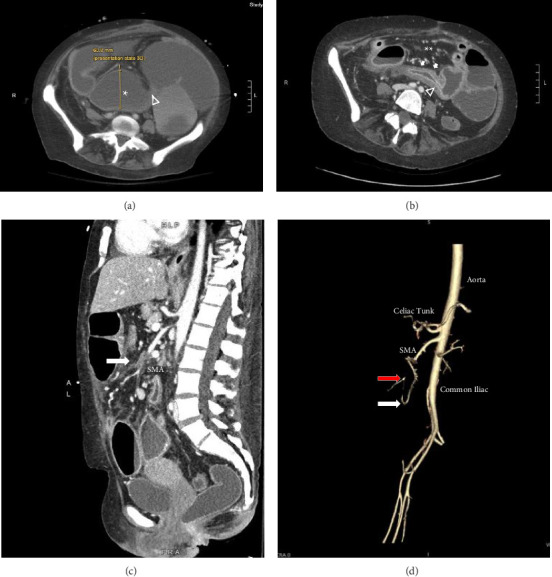
Computed tomography (CT) images of multifocal long-segment ileal and jejunal stricture secondary to superior mesenteric artery (SMA) thrombus following ileocolic resection: (a) axial CT demonstrating an ileal stricture (arrowhead) with proximal small bowel dilation (^∗^) to 60.8 mm. (b) Axial CT enterography demonstrating an ileal stricture (arrowhead) with bowel wall thickening and mural hyperenhancement (solid arrow) with adjacent vasa recta engorgement (^∗∗^). (c) Sagittal CT mesentery with (d) 3D volume-rendered imaging demonstrating the proximal point of SMA cutoff (RED arrow) and distally reconstituted branches (white arrow).

**Figure 2:**
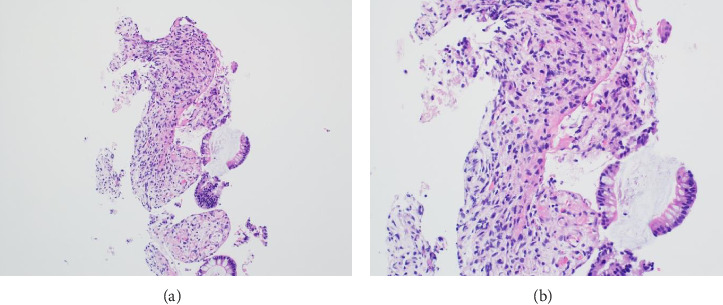
Histologic images of low and high-power views of the neoterminal ileum biopsy showing ulcer and ischemic-type changes with loss of crypts and lamina propria hyalinization and inflammation. Magnifications: (a) 100x and (b) 200x.

**Figure 3:**
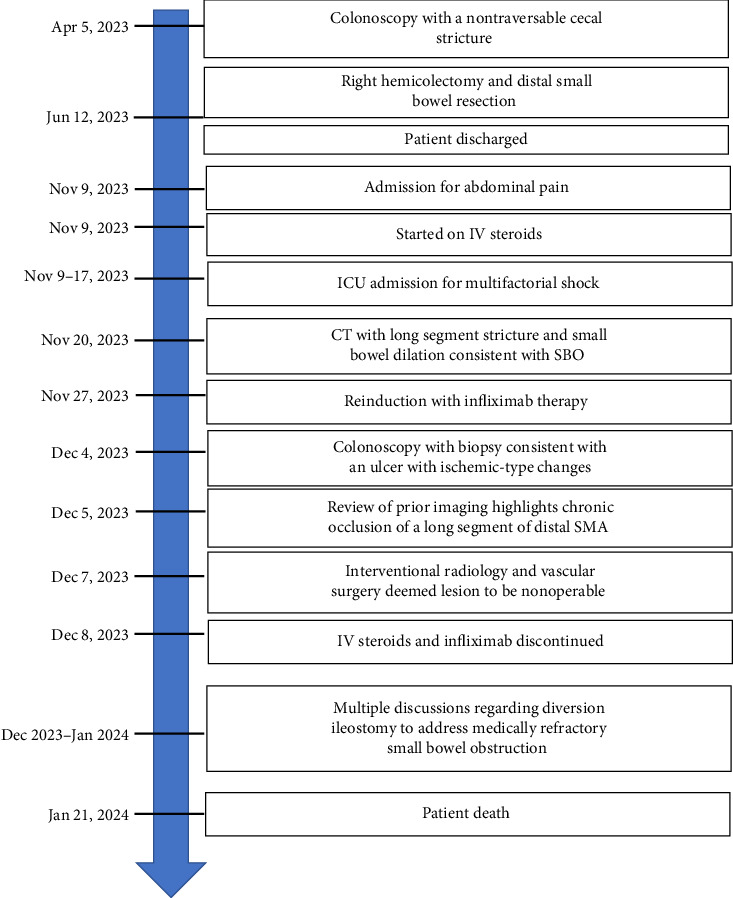
Clinical timeline of patient's hospital course.

A 46-year-old woman with long-standing short-segment ileal and colonic fibrostenotic CD (Montreal classification: A2L3B2 without perianal disease) on infliximab maintenance therapy presented to our hospital with abdominal pain and dyspnea. Five months prior to admission to our hospital, the patient experienced progressive right lower quadrant abdominal pain. A colonoscopy demonstrated a nontraversable cecal stricture. As a result, she underwent ileocolic resection (surgical and histologic margins were negative for active disease). The patient's surgery was complicated by an intraoperative duodenal injury which was repaired primarily. Postoperatively, the patient experienced multiple hospital admissions for hypotension, respiratory failure, non-ST elevation myocardial infarction, takotsubo cardiomyopathy, and severe depression resulting in poor oral intake and severe malnutrition (∼25% body weight loss, albumin 1.4 g/dL, and prealbumin 3 mg/dL) for which she was initiated on total parenteral nutrition (TPN). On admission to our hospital, the patient was hypotensive, tachycardic, and hypoxic. Computed tomography (CT) of the chest demonstrated a large pulmonary embolism with right heart strain. She was started on a heparin infusion and admitted to the intensive care unit (ICU) for management of her multifactorial shock and progressive acute hypoxic respiratory failure requiring endotracheal intubation. Her course was complicated by line-associated bloodstream infections secondary to chronic TPN use. Upon resolution of her infections, she was started on IV methylprednisolone for active CD as her CT enterography demonstrated multifocal inflammation with a 40–50 cm stricture involving her distal jejunum and ileum associated with proximal small bowel dilatation (Figures 1(a) and 1(b)) as well as possible enteroenteric fistula. A nasogastric tube was placed for decompression of small bowel obstruction (SBO). Given the prior response to infliximab and lack of other available inpatient targeted anti-inflammatory therapies, she was reinduced, after an 8-month drug holiday, with two doses of infliximab at 10 mg/kg without improvement in clinical SBO symptoms, inflammatory markers, or radiologic disease activity. Due to a lack of improvement, a colonoscopy was performed. Colonoscopy demonstrated congestion and nontraversable stenosis at the ileocolonic anastomosis preventing direct visualization. A biopsy of the neoterminal ileum stricture revealed an ulcer with ischemic-type changes (Figure 2). A review of her imaging completed during this hospitalization revealed a lack of enhancement of the lumen of the distal superior mesenteric artery (SMA) and jejunal branches (Figures 1(c) and 1(d)). Interventional radiology and vascular surgery were consulted; however, both felt the lesion was not intervenable due to the distal location of the SMA injury and the remoteness of the initial injury. IV methylprednisolone and infliximab were discontinued with the plan for surgical small bowel resection or fecal diversion when medically and nutritionally optimized due to the severity of her debility. At multiple points throughout her hospital course, diversion ileostomy was offered; however, the patient continuously declined as she was nervous about undergoing another surgery given her prior surgical complications. Notably, she never developed significant lactic acidosis or radiologic signs of bowel necrosis or perforation. Unfortunately, the patient developed septic shock secondary to aspiration pneumonia requiring transfer to the ICU where she eventually succumbed to her illness and passed away. A full timeline of the patient's medical course is summarized in Figure 3.

## 3. Discussion

We describe a case of ischemic enteritis of the small bowel initially misclassified as a manifestation of the patient's known CD. While CD is a common cause of small bowel stricture formation, strictures can be related to other etiologies, including malignancy, prior surgery, radiation, and ischemia [4]. This case underscores the importance of re-evaluating the underlying diagnosis when the presentation does not fit the typical pattern of a disease.

In this case, a re-review of prior imaging highlighted a chronic lack of small bowel perfusion from the distal SMA, which retrospectively, could be seen on the patient's imaging shortly after her hemicolectomy 6 months before her initial presentation to our hospital. The patient most likely developed an SMA thrombus resulting in ischemic enteritis and small bowel stenosis. The patient had multiple thrombotic risk factors including active small bowel inflammation, corticosteroid use, intra-abdominal surgery, and decreased ambulation after surgery. This is supported by a diagnosis of an extensive pulmonary embolus. It is also possible, although less likely, that the patient developed an iatrogenic intraoperative SMA injury. The patient's intraoperative report noted an iatrogenic tear of her duodenum, which corresponds to the region where the SMA resides within the abdominal cavity. Most SMA injuries are documented following abdominal trauma or intracardiac thrombus, whereas an iatrogenic SMA injury is rare [4, 5].

The diagnosis of ischemic enteritis was based on a convergence of clinical, radiologic, and histologic findings. Although the biopsy was obtained from the neoterminal ileum at the ileocolonic anastomosis, CT enterography showed contiguous long-segment inflammation and stricturing involving the distal jejunum and ileum, extending toward the anastomosis. Thus, the biopsy site represented the most accessible portion of a continuous process rather than an isolated or anatomically distant lesion. Ischemic-type changes on histology, coupled with imaging evidence of small bowel hypoperfusion, supported a diagnosis of ischemia.

Postischemic strictures are rare sequelae of ischemic bowel disease and are difficult to differentiate from other stricturing bowel diseases such as CD, bowel radiation, or malignancy due to overlapping clinical symptoms and histopathological findings [4, 6]. Several cases of postischemic strictures have been identified and have been associated with various etiologies, including mesenteric venous thrombosis, traumatic injury, and postoperative complications [7–9]. Given the nonspecific clinical presentation, imaging plays a crucial role in differentiation. A study examining CT features in eight cases of post-ischemic bowel strictures identified several distinguishing characteristics [6]. Postischemic strictures can develop in any segment of the bowel, but they most commonly occur in watershed areas and typically appear within weeks of an ischemic event. On imaging, these strictures often present as concentric wall thickening of medium length with moderate to high enhancement in the portal phase, exceeding that in the arterial phase [6]. In contrast, strictures associated with active CD typically exhibit stable disease localization, often involving multiple bowel segments with characteristic skip lesions predominantly within the terminal ileum [3, 6, 10]. As a result, a comprehensive approach incorporating clinical, radiographic, and histologic findings should be used to differentiate between postischemic strictures and strictures associated with CD.

This case also highlights an important learning point regarding diagnostic prioritization and therapeutic strategy. The patient's lack of clinical or radiographic improvement despite bowel rest, corticosteroids, and reinduction with infliximab highlighted a challenging clinical scenario. At the time, the presence of known CD and imaging findings consistent with active inflammation guided the therapeutic approach. However, in retrospect, the atypical location of the stricture and its resistance to standard therapies may have warranted earlier consideration of alternative, noninflammatory etiologies such as ischemia or postoperative vascular changes. In addition, while the patient had mutiple thrombotic risk factors, coagulation markers were not routinely assessed. This case represents a valuable learning opportunity, as earlier evaluation for thrombotic complications could have provided an earlier diagnosis. For similar patients with multifactorial presentations, incorporating early assessment for vascular pathology may help refine diagnostic reasoning and guide more tailored management.

In conclusion, we present a case of ischemic enteritis and stenosis due to a likely SMA thrombosis in a patient with a long-standing history of fibrostenotic CD. In the complex landscape of patient care, especially within the realm of chronic inflammatory conditions such as CD, it is a common pitfall to anchor on a prior diagnosis when a patient presents with uncommon symptoms or does not respond to conventional management [11]. While Occam's Razor, the idea that the simplest explanation is usually the correct one, serves as a useful heuristic, we must not overlook Hickam's dictum, which posits that patients can have multiple, unrelated conditions simultaneously [12]. This counterpoint to Occam's Razor serves as a crucial reminder that the reality of clinical practice often defies simplicity [12]. When faced with clinical presentations that diverge from the expected pattern, it is imperative to resist the comfort of cognitive shortcuts and instead, conduct a thorough re-evaluation from square one.

## Data Availability

The data that support the findings of this study are available on request from the corresponding author. The data are not publicly available due to privacy or ethical restrictions.
